# The thermal and electrical properties of the promising semiconductor MXene Hf_2_CO_2_

**DOI:** 10.1038/srep27971

**Published:** 2016-06-15

**Authors:** Xian-Hu Zha, Qing Huang, Jian He, Heming He, Junyi Zhai, Joseph S. Francisco, Shiyu Du

**Affiliations:** 1 Engineering Laboratory of Specialty Fibers and Nuclear Energy Materials, Ningbo Institute of Materials Technology and Engineering, Chinese Academy of Sciences, Ningbo, Zhejiang, 315201, China; 2 Center for Translational Medicine, Department of Biotechnology, Dalian Institute of Chemical Physics, Chinese Academy of Sciences, Dalian, Liaoning, 116023, China; 3 State Nuclear Power Research Institute, Beijing, 100029, China; 4 Beijing Institute of Nanoenergy and Nanosystems, Chinese Academy of Sciences, Beijing 10083, China; 5 Departments of Chemistry and Earth and Atmospheric Science, Purdue University, West Lafayette, IN 47906, USA

## Abstract

With the growing interest in low dimensional materials, MXenes have also attracted considerable attention recently. In this work, the thermal and electrical properties of oxygen-functionalized M_2_CO_2_ (M = Ti, Zr, Hf) MXenes are investigated using first-principles calculations. Hf_2_CO_2_ is determined to exhibit a thermal conductivity better than MoS_2_ and phosphorene. The room-temperature thermal conductivity along the armchair direction is determined to be 86.25~131.2 Wm^−1^ K^−1^ with a flake length of 5~100 μm. The room temperature thermal expansion coefficient of Hf_2_CO_2_ is 6.094 × 10^−6^ K^−1^, which is lower than that of most metals. Moreover, Hf_2_CO_2_ is determined to be a semiconductor with a band gap of 1.657 eV and to have high and anisotropic carrier mobility. At room temperature, the Hf_2_CO_2_ hole mobility in the armchair direction (in the zigzag direction) is determined to be as high as 13.5 × 10^3^ cm^2^V^−1^s^−1^ (17.6 × 10^3^ cm^2^V^−1^s^−1^). Thus, broader utilization of Hf_2_CO_2_, such as the material for nanoelectronics, is likely. The corresponding thermal and electrical properties of Ti_2_CO_2_ and Zr_2_CO_2_ are also provided. Notably, Ti_2_CO_2_ presents relatively lower thermal conductivity but much higher carrier mobility than Hf_2_CO_2_. According to the present results, the design and application of MXene based devices are expected to be promising.

Because two-dimensional (2D) materials exhibit many novel electronic and thermal properties that differ from those of their bulk counterparts, these materials have received considerable attention over the past two decades. For example, the adoption of 2D materials in place of traditional bulk materials for the next generation of electronic devices has recently been demonstrated as a potentially practical strategy^1^. Since the size of electronic devices has been continuously decreasing over the past twenty years during the development of highly integrated electronic components, efficient heat dissipation, a moderate electronic band gap and a reasonably high carrier mobility^2^ have become equally important properties in determining the performance of electronic devices. Graphene, a well-known 2D carbon material^3^, has been demonstrated to possess a high thermal conductivity and high charge carrier mobility^4^, which have strongly encouraged ongoing research into expanding its applicability as a material for thermal conduction or electronic devices. Many other similar classes of 2D materials have also been fabricated as candidate materials for nanoelectronics, including monolayers of h-BN^5^, phosphorene^2^,^6^,^7^,^8^, and transition-metal dichalcogenides^9^,^10^. In-depth investigations of these monolayers have been conducted and have led to several potential applications^11^. However, additional treatment on these 2D materials is generally required, such as structural modification, composition, or application of external fields^11^ for their practical application in highly integrated electronic components. The reasons for these requirements are as follows: graphene is a zero-band-gap semiconductor in nature and thus its band gap needs to be opened^12^; monolayer h-BN^13^ has an excessively large band gap (5.5 eV); the thermal conductivities of monolayer MoS_2_ and phosphorene [(34.5 ± 4) and 11.8 Wm^−1^K^−1^, respectively] are not impressive in terms of heat dissipation^7^,^14^. Moreover, the carrier mobility of MoS_2_ is not ideally high^15^, and phosphorene is prone to chemical degradation upon exposure to ambient conditions^16^. In other words, difficulties may occur if these materials are directly applied in highly integrated electronic components considering their intrinsic properties. Therefore, the discovery of a desirable 2D semiconducting material with a moderate band gap, satisfactory intrinsic thermal conductivity and high carrier mobility remains a primary goal of research in physics and materials science.

Recently, a new family of 2D transition-metal carbides and nitrides called “MXene”^17^,^18^,^19^,^20^,^21^,^22^ has been fabricated by selective etching of “A” from M_n+1_AX_n_ phases (where M is an early transition metal, A is an A-group element, X is C and/or N, and n = 1, 2, or 3). Since M_n+1_AX_n_ materials represent a large family that consists of more than 60 members^23^, the corresponding MXenes inherit versatile configurations. To date, the following MXenes have been synthesized by exfoliation of the corresponding M_n+1_AX_n_ phases and relatives with a hydrofluoric acid treatment: Ti_3_C_2_^17^, Ti_2_C, Ta_4_C_3_, TiNbC, (V_0.5_, Cr_0.5_)_3_C_2_, Ti_3_CN^18^, Z_3_C_2_^24^, Mo_2_TiC_2_, Mo_2_Ti_2_C_3_, Cr_2_TiC_2_^25^, Nb_4_C_3_^22^, Mo_2_C^26^, Nb_2_C and V_2_C^19^. The as-synthesized MXenes are typically functionalized by -O, -OH and -F groups. Naguib *et al.*^21^ have published a review of these materials in which they denote functionalized MXenes as M_n+1_X_n_T_x_, with T standing for the surface-terminating group. Both theoretical and experimental results have demonstrated that MXenes have potential applications in hydrogen storage^27^, lithium-ion batteries (LIBs)^28^,^29^, supercapacitors^30^,^31^, adsorbents^32^,^33^ and electronic devices^34^,^35^. As the attention on this new class of MXene materials grows, gaining deeper insight into their basic physical properties becomes more important. However, only few studies^36^,^37^ have been published in the literature on the thermal properties and carrier mobility of MXene materials.

In this work, the electronic band gap, thermal properties and carrier mobility of three oxygen-functionalized MXenes, Ti_2_CO_2_, Zr_2_CO_2_ and Hf_2_CO_2_, are predicted via theoretical calculations. Oxygen-functionalized MXenes are chosen in this work for their current applicability compared to those functionalized by -F and -OH groups^29^,^38^,^39^,^40^,^41^ and for their higher thermodynamic stability^38^. Based on the results presented herein, Hf_2_CO_2_ is unexpectedly determined to possess a moderate band gap, a thermal conductivity better than MoS_2_ and phosphorene, and a high carrier mobility comparable to phosphorene, which indicates that this material may have extensive potential applications in nanoelectronics. For this reason, the major part of this work focuses on the thermal conductivity and electrical properties of Hf_2_CO_2_. In addition, the specific heat and thermal expansion coefficient of Hf_2_CO_2_ are provided as well. The corresponding values for Ti_2_CO_2_ and Zr_2_CO_2_ are also examined and discussed for comparison to the results for Hf_2_CO_2_. According to the computational results, the thermal conductivity increases with increasing atomic number of M among M_2_CO_2_ (M = Ti, Zr, Hf) MXenes and all the three MXenes present high carrier mobilities. Moreover, Ti_2_CO_2_ are found to possess much lower thermal conductivity and higher hole mobility than Zr_2_CO_2_ and Hf_2_CO_2_. The results for Ti_2_CO_2_ and Zr_2_CO_2_ are mainly supplied in the Supplementary Information.

## Results

**Figure 1:**
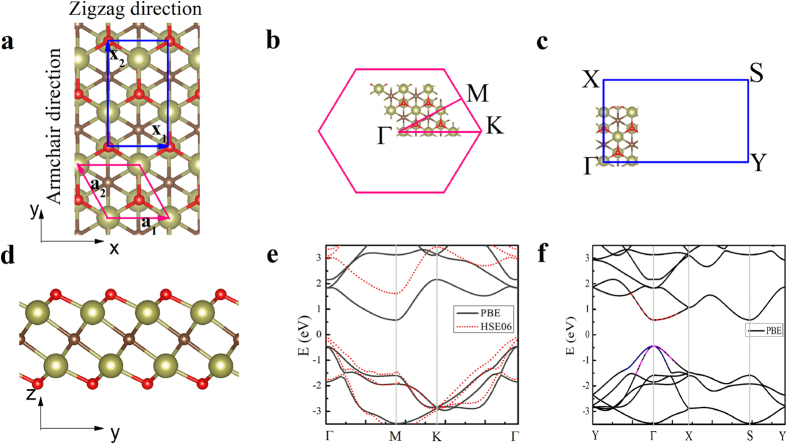
Structure and electronic band structure of Hf_2_CO_2_. (**a**) Top-view of the Hf_2_CO_2_ structure; the hexagonal unit cell and orthorhombic cell are circled in pink and blue boxes, respectively; the *x*- (*y*-) axis corresponds to the Hf_2_CO_2_ zigzag (armchair) direction. (**b**) The Brillouin zone (BZ) of the hexagonal unit cell; the ΓΜ (ΓΚ) direction in reciprocal space corresponds to the Hf_2_CO_2_ armchair (zigzag) direction in real space. (**c**) The BZ of the Hf_2_CO_2_ orthorhombic cell. (**d**) The side-view of Hf_2_CO_2_. (**e**) The electronic band structure of Hf_2_CO_2_. The band gap is increased using the HSE06 correction. The Fermi level is located at 0 eV. (**f**) The Hf_2_CO_2_ electronic band structure based on the orthorhombic cell. The valance band maximum (VBM) and conduction band minimum (CBM) are denoted by colored lines.

The geometries and electronic properties of Hf_2_CO_2_ are investigated using DFT calculations. The Hf_2_CO_2_ MXene shows a hexagonal lattice with its space group of 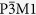
(No. 164). Similar to graphene and other 2D hexagonal materials^11^, the MXene possesses two high-symmetry routes namely the armchair and zigzag directions. Seen from the top-view as shown in Fig. 1a, the x-axis coincides with the zigzag direction, and the y-axis is parallel to the armchair direction in our model. Moreover, based on the hexagonal lattice, the ΓΜ route in the Brillouin zone (BZ) corresponds to the armchair direction in the real-space, and the ΓΚ vector is along the zigzag direction as presented in Fig. 1b. The periodicities along the two high symmetry directions can be more clearly displayed with an orthorhombic lattice as shown in Fig. 1c. Evidently, the basis vector along the zigzag direction is smaller than that in the armchair direction, whose length is determined to be 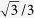 of that for the latter. As seen from the side-view in Fig. 1d, a central carbon monolayer is sandwiched between two Hf layers, and the oxygen layer is directly projected to the bottom Hf layer on both sides. The stable structure is similar to those of other stable oxygen-functionalized M_2_CO_2_ (M is a transition metal) MXenes^42^, including Ti_2_CO_2_ and Zr_2_CO_2_, whose geometries differ only slightly from Hf_2_CO_2_ in bond lengths and lattice constants. To be more explicitly, the lattice parameters, layer thicknesses, bond lengths and atomic charges for these three MXenes are given in Table 1. Evidently, the Zr_2_CO_2_ shows the largest atomic charges, and the atomic charges in Ti_2_CO_2_ are significantly smaller, which reflects that Zr_2_CO_2_ possesses stronger bonds than Ti_2_CO_2_. The lattice parameter and unit cell volume of Hf_2_CO_2_ appear to be smaller than Zr_2_CO_2_ despite that Hf has a larger atomic radius than Zr, showing the strength of the bonds may be stronger in Hf_2_CO_2_ compared to that in Zr_2_CO_2_. Therefore, the mechanical strength increases with the increasing atomic number of M among these three MXenes^41^. Figure 1e depicts the electronic energy band of Hf_2_CO_2_. From GGA calculations, Hf_2_CO_2_ is an indirect semiconductor with a band gap of 1.021 eV, which agrees well with previous findings^42^. After HSE06 correction, this band gap increases to 1.657 eV, which is comparable to those of monolayer MoS_2_ and phosphorene^2^,^9^. Since the HSE method is demonstrated to yield a 0.3 eV mean absolute error smaller than semiconductors’ band gaps^43^, the true band gap for Hf_2_CO_2_ may reach 1.957 eV. Figure 1f shows the electronic energy band based on the orthorhombic lattice, in which the conduction-band minimum (CBM) is folded to the Γ point. Two sub-bands overlap at the valance-band maximum (VBM), with an energy difference of only 0.6 meV at the Γ point (the corresponding difference at the Γ point is 1.8 meV in the case of Ti_2_CO_2_, and 0.3 meV for Zr_2_CO_2_). All of the M_2_CO_2_ (M = Sc, Ti, Zr, Hf, V, Nb, Ta, Cr, Mo, W) electronic band structures based on GGA calculations are provided in Fig. S1. The M_2_CO_2_ (M = Sc, Ti, Zr, Hf, W) MXenes are found to be semiconductors, which is consistent with our previous work^41^; their electronic energy bands are corrected by HSE06 functional. Due to its semiconducting behavior, the electronic thermal conductivity in Hf_2_CO_2_ is negligible.

**Figure 2:**
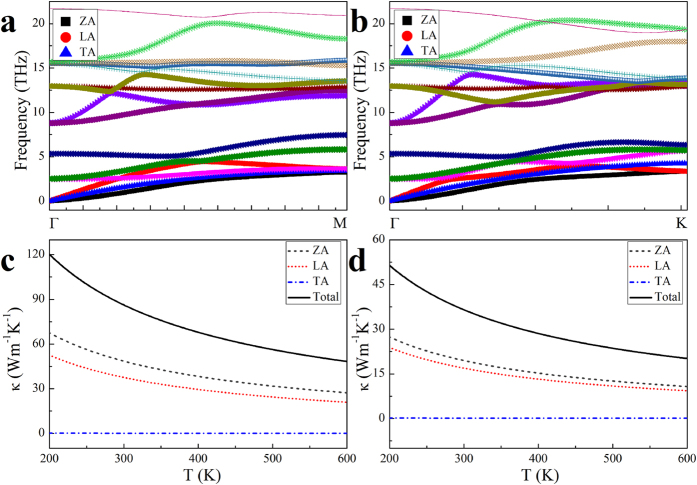
Phonon dispersions and thermal conductivities along the armchair (ΓΜ) and zigzag (ΓΚ) directions. (**a**) The phonon dispersion of Hf_2_CO_2_ along the armchair direction. The out-of-plane acoustic (ZA), longitudinal acoustic (LA) and transversal acoustic (TA) modes are denoted with black squares, red circles and blue triangles, respectively. (**b**) The phonon dispersion of Hf_2_CO_2_ along the zigzag direction. (**c**) The temperature dependence of the Hf_2_CO_2_ thermal conductivity along the armchair direction. The ZA, LA and LA mode contributions to the thermal conductivity are denoted with grey dashed, red dotted and blue dash-dotted lines, respectively. (**d**) The temperature dependence of the Hf_2_CO_2_ thermal conductivity along the zigzag direction.

The Hf_2_CO_2_ phonon dispersions along the armchair (ΓΜ) and zigzag (ΓΚ) directions are shown in Fig. 2a,b, respectively. The out-of-plane acoustic (ZA), longitudinal acoustic (LA) and transversal acoustic (TA) modes are denoted with black squares, red circles and blue triangles, respectively. The ZA mode nearly coincides with the TA mode in the armchair direction, differing from that along the zigzag direction. From the phonon dispersion, the group velocities of the acoustic modes in the armchair direction are determined as *ν*_ZA_ = 1.826 × 10^3^ ms^−1^, *ν*_TA_ = 1.919 × 10^3^ ms^−1^ and *ν*_LA_ = 2.065 × 10^3^ ms^−1^, and the corresponding group velocities in the zigzag direction are *ν*_ZA_ = 1.641 × 10^3^ ms^−1^, *ν*_ TA_ = 2.075 × 10^3^ ms^−1^ and *ν*_LA_ = 1.656 × 10^3^ ms^−1^. The value of Grüneisen parameter *γ*_j_ representing MXene anharmonic effect is determined from the phonon dispersions using optimized (a = 1.00 *a*_0_ where *a*_0_ is the optimized hexagonal lattice parameter in the plane parallel to BZ) and strained (a = 0.99*a*_0_ and a = 1.01*a*_0_) configurations. From the calculations with the conventional method^44^, *γ*_j_ for the acoustic modes of Hf_2_CO_2_ are *γ*_ZA_ = −0.164, *γ*_TA_ = −1.254 and *γ*_LA_ = 1.032 in the armchair direction and *γ*_ZA_ = −0.263, *γ*_TA_ = −0.916 and *γ*_LA_ = 1.240 in the zigzag direction. Evidently, the group velocities are larger and Grüneisen parameters are smaller for the ZA and LA modes in the armchair direction than those in the zigzag direction, and the TA mode shows the opposite trend. The values of 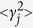 as well as *γ*_*j*_ and *υ*_*j*_ used for calculating the thermal conductivities of M_2_CO_2_ (M = Ti, Zr, Hf) MXenes are given in Table 2. Generally, *υ*_*j*_ and *γ*_*j*_ decrease with the increasing atomic number of M among M_2_CO_2_ (M = Ti, Zr, Hf) MXenes. The Hf_2_CO_2_ thermal conductivities along the armchair and zigzag directions are calculated using Equation (1); the results are depicted in Fig. 2c,d, respectively. A flake length of 5 μm is adopted in both cases. According to the figures, the Hf_2_CO_2_ thermal conductivity is strongly anisotropic due to the anisotropic group velocities and Grüneisen parameters as indicated above. This can be qualitatively understood from the atomic arrangement of Hf_2_CO_2_. As seen in Fig. 1a, the projections of some Hf-O and Hf-C bonds on the material plane are parallel to the armchair direction, whereas no projections of any bonds can coincide with the zigzag direction, which causes different interatomic force in vibration propagating along different directions. Consequently, the phonon frequencies and group velocities are different between the sound waves propagating along the armchair and zigzag directions. Moreover, the different bonding strengths projected to the two directions also causes different anharmonic effects, leading to anisotropic Grüneisen parameters. Therefore, the anisotropy of the thermal conductivity can essentially be attributed to the atomic configuration of the MXene. In the armchair direction, the room temperature thermal conductivity is determined to be 86.25 Wm^−1^K^−1^, the corresponding value in the zigzag direction is only 42.3% of the former. In both directions, the thermal conductivity is mainly contributed by ZA and LA modes, with the contribution from ZA mode is slightly higher. For example, in the armchair direction, the ZA and LA modes’ contributions to the thermal conductivity at room temperature are 48.55 and 37.58 Wm^−1^K^−1^, respectively. However, the large contribution of ZA mode to thermal conductivity is absent in Ti_2_CO_2_ and Zr_2_CO_2_, as shown in Figs S2 and S3, respectively. For these two M_2_CO_2_ (M = Ti, Zr) MXenes, their conductivities are mainly contributed by TA and LA modes.

**Figure 3:**
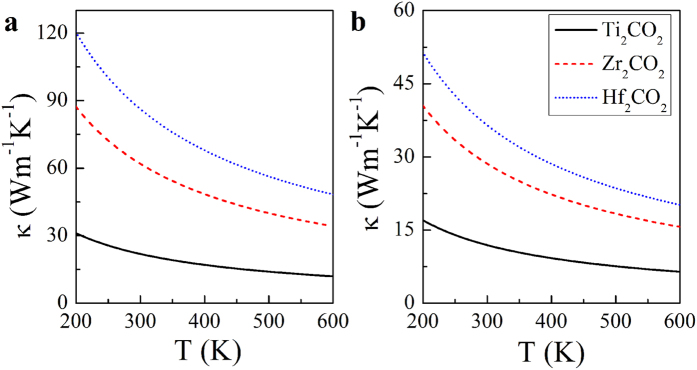
The temperature dependence of the thermal conductivities of the M_2_CO_2_ (M = Ti, Zr, Hf) MXenes. (**a**) The temperature dependence of the thermal conductivities of the M_2_CO_2_ (M = Ti, Zr, Hf) MXenes along the armchair direction. The Ti_2_CO_2_, Zr_2_CO_2_ and Hf_2_CO_2_ thermal conductivities are denoted in black solid, red dashed and blue dotted lines, respectively. (**b**) The temperature dependence of the thermal conductivities of the M_2_CO_2_ (M = Ti, Zr, Hf) MXenes along the zigzag direction.

For comparison, we plot the thermal conductivities of all the three M_2_CO_2_ (M = Ti, Zr, Hf) MXenes in Fig. 3 based on a 5 μm flake length. Figure 3a shows the thermal conductivities in the armchair direction, and Fig. 3b depicts those in the zigzag direction. Evidently, all the three MXenes present strong anisotropic thermal conductivities, and the ratios between the zigzag and armchair thermal conductivities of M_2_CO_2_ (M = Ti, Zr, Hf) MXenes are 54.4%, 46.2% and 42.3% respectively. One can also find that the thermal conductivity increases significantly with increasing atomic number of M in both directions. As an example, the room temperature thermal conductivities of Ti_2_CO_2_ in armchair direction is determined to be 21.88 Wm^−1^K^−1^, while the corresponding values in Zr_2_CO_2_ and Hf_2_CO_2_ increase to 61.93 and 86.25 Wm^−1^K^−1^, respectively. As to the zigzag direction, the room temperature values for M_2_CO_2_ (M = Ti, Zr, Hf) MXenes follows the similar trend, which are 11.91, 28.59, 36.51 Wm^−1^K^−1^ respectively. This can be explained by the enhanced mechanical strengths of MXenes with increasing atomic number of M^41^. The thermal conductivity of Hf_2_CO_2_ in armchair direction is higher than those of many well-known thermal conductive materials, such as pure iron^45^, suggesting that Hf_2_CO_2_ may have satisfactory performance in heat conduction as a 2D oxide material. Additionally, it is worthy of accentuating that the thermal conductivity of Hf_2_CO_2_ is higher than those of MoS_2_ and phosphorene which are well known promising semiconducting 2D materials for nanoelectronics.

**Figure 4:**
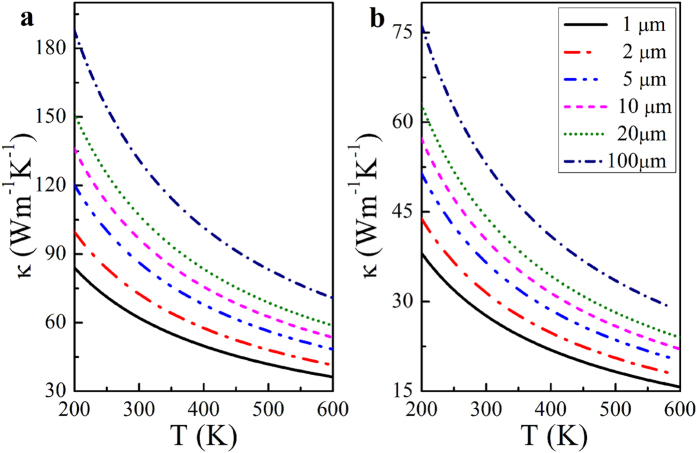
The temperature dependence of Hf_2_CO_2_ thermal conductivity with varying flake lengths. (**a**) The temperature dependence of the thermal conductivity with varying flake lengths in the armchair direction. The thermal conductivity for flake lengths of 1, 2, 5, 10, 20 and 100 μm are denoted by black solid, red dash-dotted, blue dashed-dotted, magenta dashed, olive dotted and navy dash-dotted lines, respectively. (**b**) The temperature dependence of the Hf_2_CO_2_ thermal conductivity with varying flake lengths in the zigzag direction.

Because of boundary scattering, the thermal conductivity is dependent upon the flake length*d*. The theoretical thermal conductivity of Hf_2_CO_2_ with flake lengths from 1 to 100 μm in the armchair and zigzag directions are shown in Fig. 4a,b, respectively. The thermal conductivity increases monotonically with increasing flake length in both directions. Moreover, the thermal conductivity is more sensitive to the flake length at low temperatures. At room temperature, the thermal conductivity in the zigzag direction increases from 27.63 to 53.03 Wm^−1^K^−1^, for a flake-length increase from 1 to 100 μm. With the same range of the flake length, the room temperature thermal conductivity in the armchair direction ranges from 62.12 to 131.2 Wm^−1^K^−1^. These results further confirm the capability of Hf_2_CO_2_ for heat dissipation if used in an electronic device.

**Figure 5:**
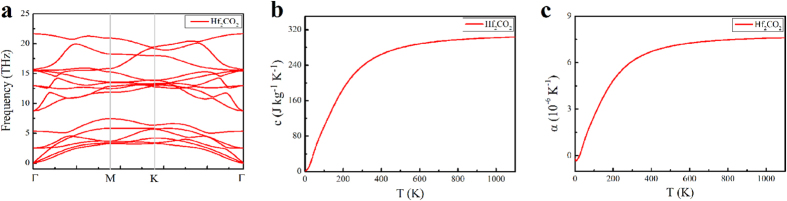
The phonon dispersion, specific heat and thermal expansion coefficient of Hf_2_CO_2_. (**a**) The phonon dispersion of Hf_2_CO_2_ in the BZ. (**b**) The temperature dependence of Hf_2_CO_2_ specific heat. (**c**) The temperature dependence of the Hf_2_CO_2_ thermal expansion coefficient.

The specific heat and thermal expansion coefficient are also studied from the phonon dispersion for the hexagonal BZ of Hf_2_CO_2_ (Fig. 5a). The specific heat and thermal expansion coefficient are presented in Fig. 5b,c, respectively. The results show that the specific heat and thermal expansion coefficient increase with increasing temperature and that the room-temperature values are 0.238 × 10^3^ Jkg^−1^K^−1^ and 6.094 × 10^−6^ K^−1^, respectively. The room-temperature thermal expansion coefficient is lower than that for most metals, such as 16.50 × 10^−6^ K^−1^ for bulk copper. The low thermal expansion coefficient is another advantage of Hf_2_CO_2_ in applications requiring structural stability at varying temperatures such as in electronic devices.

In the calculation of carrier mobility, the orthorhombic cell is adopted, which makes the elastic modulus along the transport direction well defined. Moreover, it facilitates the determination of the electron effective masses in the *x*- and *y*-directions. Before predicting the carrier mobility of Hf_2_CO_2_, we perform a benchmark calculation on the electron mobility of phosphorene. The room-temperature electron mobility of phosphorene is determined to be 1.387 × 10^3^ and 0.177 × 10^3^ cm^2^V^−1^s^−1^ along the armchair and zigzag directions, respectively, which is in good agreement with the results of previous works^2^,^8^. In this study, both the electron and hole mobilities of Hf_2_CO_2_ are evaluated and the results are listed in Table 3. The electron mobilities are found highly anisotropic, and the values along the zigzag (*x*-) and armchair (*y*-) directions are 0.329 × 10^3^ and 0.077 × 10^3^ cm^2^V^−1^s^−1^, respectively, at room temperature. As to the calculations for hole mobilities, one should notice that two quasi-degenerated sub-bands appear at the VBM as shown in Fig. 1f. Hence, the hole mobilities of both sub-bands should be calculated and the total hole mobility can be estimated as the statistical average of the two sub-bands on the basis of Boltzmann’s distribution. For clear elaboration, we denote the sub-band with a higher (lower) energy at the Γ point as the “upper” (“lower”) band in Table 3. From the “upper” sub-band (denoted in blue in Fig. 1f), the hole mobilities are determined to be 0.924 × 10^3^ and 26.0 × 10^3^ cm^2^V^−1^s^−1^ along the Hf_2_CO_2_ zigzag and armchair directions, respectively. Correspondingly, the hole mobilities of the “lower” band (denoted in megaton in Fig. 1f) are calculated as 34.3 × 10^3^ and 1.00 × 10^3^ cm^2^V^−1^s^−1^ along the zigzag and armchair directions of Hf_2_CO_2_, respectively. The high hole mobilities are mainly caused by the small hole effective mass and low deformation potential constant, as described in the table. Notably, the effective masses and deformation potential constants for the two sub-bands are close to each other in value, but their directions appear to be opposite. For the “upper” sub-band, the hole effective mass and deformation potential constant in the zigzag direction are both approximately threefold higher than those in the armchair direction, causing the highly anisotropic hole mobility (approximately twenty-eight-fold higher in the armchair direction). However, the sequence of hole mobilities along the two directions for the “lower” sub-band are totally reversed reflected by the seemingly “exchanged” effective masses and deformation potential constants. Consequently, the average hole mobilities exhibit only slight anisotropy, which are 17.6 × 10^3^ and 13.5 × 10^3^ cm^2^V^−1^s^−1^ along the Hf_2_CO_2_ zigzag and armchair directions, respectively. Evidently, the predicted hole mobility is much higher than the electron mobility, which can be ascribed to the lighter effective masses and smaller deformation potential constants of the holes as presented in Table 3. The predicted high carrier mobilities are much higher than that of monolayer MoS_2_^15^, and they are comparable to the hole mobility of monolayer phosphorene^2^ along the zigzag direction. Moreover, the oxygen functionalized Hf_2_CO_2_ MXene may show higher stability than phosphorene. Thus, the current results indicate that Hf_2_CO_2_ may perform well as a material in nanoelectronics.

**Figure 6:**
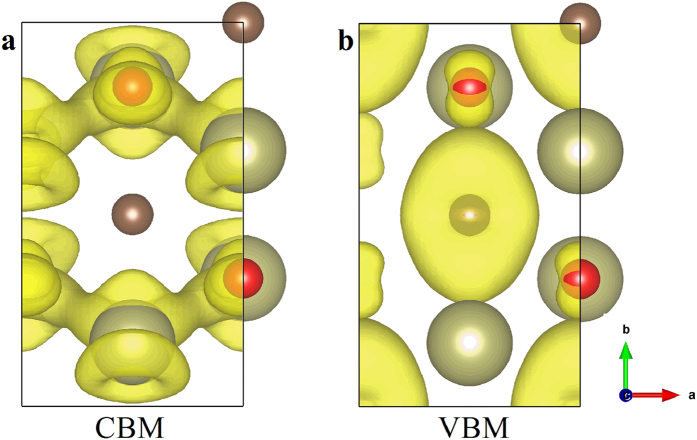
The electronic wavefunctions of the CBM and VBM for Hf_2_CO_2_ based on the orthorhombic cell. (**a**) CBM. (**b**) VBM.

Different from the anisotropy of the phonon thermal conductivity caused by the MXene atomic configurations, the carrier mobility is mainly related to the electronic wavefunctions at the VBM and CBM. Therefore, to understand the difference in anisotropy between the electron and hole mobilities, the electronic wavefunctions for the CBM and VBM (the sum of “upper” and “lower” sub-bands) on the basis of the orthorhombic cell are plotted in Fig. 6. From the figure, the electronic wavefunctions of CBM are mainly contributed by the hafnium and oxygen atoms, and the wavefunctions show clear delocalization feature along the zigzag direction but there exist nodes along the armchair direction. Thus, the electron mobility is much larger in the zigzag direction. Regarding to the electronic wavefunctions for the VBM as shown in Fig. 6b, they are mainly from the carbon atoms and nodes can be found along both directions, which is different from the CBM. Accordingly, the hole mobility of the Hf_2_CO_2_ present only slight anisotropy.

The carrier mobilities of Ti_2_CO_2_ and Zr_2_CO_2_ are also calculated, as shown in Tables S1 and S2, respectively. It is noteworthy that Ti_2_CO_2_ presents much higher hole mobility compared with Zr_2_CO_2_ and Hf_2_CO_2_. The statistical average hole mobility of Ti_2_CO_2_ is predicted to be 33.6 × 10^3^ and 26.6 × 10^3^ cm^2^V^−1^s^−1^ along the zigzag and armchair directions, respectively, which are approximately two fold greater than that of Hf_2_CO_2_. The predicted high hole mobilities are well consistent with the experimental findings for Ti_2_CT_x_ MXene^37^, in which the room temperature carrier mobility is measured to be in order of 10^4^ cm^2^V^−1^s^−1^. The consistency between our work and experimental measurement further indicates the reliability of our calculations. In addition, Khazaei *et al.* have demonstrated Ti_2_CO_2_ possesses a large Seebeck coefficient 46. This implies that Ti_2_CO_2_ may be applicable as thermoelectric materials^47^ because of the low thermal conductivity and high carrier mobility determined here. Furthermore, Ti_2_CO_2_ may be preferred over Hf_2_CO_2_ for use in electronic devices in cases heat dissipation is not the major concern.

## Discussion

The combination of the results for the carrier mobilities with those for the thermal properties suggests that Hf_2_CO_2_ is a good choice for nanoelectronics applications. Because of the limited data currently available, further research should be conducted on the synthesis of M_2_CO_2_ (M = Zr, Hf) monolayers, and their intrinsic thermal and electrical properties should be experimentally measured. Considering the successful fabrication of Ti_2_CT_2_ (T = -O, -F, -OH) and the existence of MAX phases M_2_AC (M = Zr, Hf; A = In, Tl, Sn, Pb, S)^48^, there is a great anticipation on the synthesis of M_2_CO_2_ (M = Zr, Hf) using the reported preparation methods such as etching of their parental MAX phase and heat treatment on hydroxyl functionalized MXenes. We look forward to more findings reported for these three MXenes from the forthcoming experimental studies.

In summary, the thermal and electrical properties of Hf_2_CO_2_ are investigated. The Hf_2_CO_2_ band gap is determined to be 1.657 eV. The thermal conductivity of Hf_2_CO_2_ in the armchair direction at room temperature is predicted to be 86.25 Wm^−1^K^−1^ with a flake length of 5 μm; this thermal conductivity is higher than those of pure iron and some other well known two dimensional materials including MoS_2_ and phosphorene. Moreover, the Hf_2_CO_2_ thermal conductivity is anisotropic with the thermal conductivity in the zigzag direction only 42.3% of that in the armchair direction at room temperature. In addition, the thermal expansion coefficient of Hf_2_CO_2_ is lower than that of most metals. The carrier mobility of Hf_2_CO_2_ is also predicted, with consideration of electron-phonon coupling. The room-temperature hole mobility in the armchair (zigzag) direction is calculated to be as high as 13.5 × 10^3^ cm^2^V^−1^s^−1^ (17.6 × 10^3^ cm^2^V^−1^s^−1^). Therefore, Hf_2_CO_2_ can be considered as candidate 2D materials for the design of next-generation electronic devices. The carrier mobility of Ti_2_CO_2_ is determined to be two fold higher than that of Hf_2_CO_2_ while the thermal conductivity is much lower. According to the current results, Ti_2_CO_2_ can be considered as candidate 2D thermoelectric materials and it may also be a better option than Hf_2_CO_2_ for nanoelectronics if good heat dissipation can be achieved in a device. Finally, options for further explorations of MXenes are raised on the basis of the results from the present work.

## Methods

The calculated structures and electronic properties are determined on the basis of first-principles density functional theory implemented in the plane-wave VASP code^49^. The generalized gradient approximation (GGA) of the Perdue-Burke-Ernzerhof (PBE)^50^ scheme is adopted for the exchange-correlation functional. To obtain a more reliable band gap, the Heyd-Scuseria-Ernzerhof (HSE06)^51^,^52^ hybrid functional is utilized to calculate the electronic energy bands of M_2_CO_2_ (M = Ti, Zr, Hf) hexagonal unit cells. The projected augmented wave (PAW) approach^53^ is employed for pseudopotentials; the plane-wave cutoff energy is chosen to be 500 eV. The conjugate gradient^54^ method is applied for structural optimization, and the system is relaxed until the forces on each atom are less than 1.0 × 10^−4^ eV/atom. To eliminate neighboring layer interaction, a 25-Å vacuum layer parallel to the surface layer is used. During optimization, a 12 × 12 × 1 k-points mesh is sampled in the Brillouin zone (BZ), and a 60 k-points grid is applied for plotting the electronic energy band. To investigate the atomic charge of each atom in the investigated MXenes, the Bader charge analysis^55^ based on a 180 × 180 × 1 mesh is adopted. All of the structures are visualized using the VESTA code^56^.

The thermal properties, including the phonon thermal conductivity, specific heat, and thermal expansion coefficient, are calculated from the phonon dispersion of a hexagonal unit cell, as circled in the pink rhombus in Fig. 1a. A 120 k-points grid is employed for plotting the phonon dispersion for various directions and the entire BZ. The Phonopy software^57^ combined with the VASP code is utilized for phonon dispersion calculations. The theoretical calculation is performed with the density functional perturbation theory (DFPT)^58^, and a 6 × 6 × 1 k-points mesh based on a 4 × 4 × 1 supercell is adopted for calculating the dynamical matrix. The phonon band connections are estimated from eigenvectors and the phonon band is determined considering band crossings implemented in the Phonopy software. The phonon thermal conductivity is calculated within the framework of Klemens’ theory^59^,^60^:


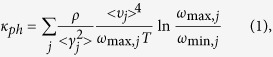


where T is temperature, 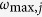 and 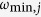 are the maximum and minimum circular frequency of each *j*^*th*^ branch. *υ*_*j*_ represents the group velocity along the temperature gradient. Due to the finite flake length *L*, the term of 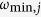 is redefined as: 
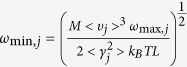
60, with *M* being the mass of the MXene unit cell. *k*_*B*_ is the Boltzmann constant. *γ*_*j*_ is the average value of the *j*^*th*^ branch Grüneisen parameter, and 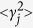 in Equation (1) is estimated by 
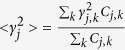
59. Variable*ρ* represents the mass density. For our hexagonal lattices, mass density is calculated as 
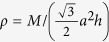
, where *a* is the lattice parameter in the *xy* plane, and *h* is the layer thickness. The value of *h* is calculated by the distance between two neighboring carbon bilayers of M_2_CO_2_ MXene, which is similar to the calculation of graphene^61^. To accurately describe the interlayer interaction of the bilayers, a damped van der Waals (VDW) correction (DFT-D2)^62^ is adopted. The flake length, *L*, which ranges from 1 to 100 μm, approaching the experimental results^31^ are considered. The thermal conductivities along the armchair and zigzag directions are both investigated, similar to the previous study conducted for graphene monoxide^63^. The method for thermal conductivity is verified by calculating the thermal conductivity of graphene (4755.6 Wm^−1^K^−1^ based on a 5 μm flake length at room temperature). The predicted values are consistent very well with the experimental result^4^. The specific heat^64^ and thermal expansion coefficient^44^,^65^ are calculated according to existing methods and our previous work.

The carrier mobility is calculated according to Equation (2), considering electron-phonon coupling^2^,^8^,^66^.


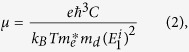


here 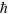 is the reduced Plank’s constant, respectively. 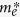 is the carrier effective mass along the transport direction; *m*_*d*_ is determined by 
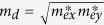
, where 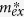 and 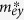 are the effective mass along the *x*- and *y*-directions, respectively; 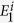 is the deformation potential constant of the valance-band maximum for holes or conduction-band minimum for electrons along the transport direction, calculated by 
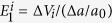
 with Δ*V*_*i*_ as the energy change of the *i*^*th*^ energy band under a small lattice variation Δ*a* and *a*_0_ as the lattice constant along the transport direction; *C* is the elastic modulus along the transport direction, determined by extrapolation based on the relationship of *C* (Δ*a*/*a*)^2^/2 = (*E* − *E*_0_)/*S*_0_, where (*E* − *E*_0_) is the change of the total energy under a varying lattice constant with a small step size 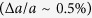 and *S*_0_ is the area of the lattice in the *xy* plane.

## Additional Information

**How to cite this article**: Zha, X.-H. *et al.* The thermal and electrical properties of the promising semiconductor MXene Hf_2_CO_2_. *Sci. Rep.* **6**, 27971; doi: 10.1038/srep27971 (2016).

## Supplementary Material

Supplementary Information

## Figures and Tables

**Table 1:** The lattice parameters, layer thicknesses, bond lengths and atomic charges for M_2_CO_2_ (M = Ti, Zr, Hf) MXenes.

MXenes	a (Å)	*h* (Å)	Bond length (Å)		Atomic charge		
			M-C	M-O	M	C	O
Ti_2_CO_2_	3.036	6.883	2.187	1.978	1.732	−1.497	−0.981
Zr_2_CO_2_	3.310	6.191	2.368	2.120	2.238	−1.954	−1.258
Hf_2_CO_2_	3.266	6.029	2.333	2.103	2.079	−1.848	−1.151

**Table 2:** Parameters for calculating the thermal conductivities of M_2_CO_2_ (M = Ti, Zr, Hf) MXenes.

MXenes		*υ*_*j*_ (10^3 ^ms^−1^)			*γ*_*j*_			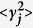		
		ZA	TA	LA	ZA	TA	LA	ZA	TA	LA
Ti_2_CO_2_	Armchair	2.566	2.666	2.974	−5.348	4.374	1.737	4088	20.42	5.379
	Zigzag	2.252	2.974	2.279	−4.457	4.317	2.252	4089	19.83	6.705
Zr_2_CO_2_	Armchair	2.379	2.518	2.715	−0.573	2.432	1.231	19.79	6.155	2.639
	Zigzag	2.113	2.640	2.136	−2.008	3.039	1.433	298.1	10.13	2.911
Hf_2_CO_2_	Armchair	1.826	1.919	2.066	−0.164	−1.254	1.032	1.256	1382	2.088
	Zigzag	1.641	2.075	1.656	−0.263	−0.916	1.240	2.316	1392	2.391

**Table 3:** Hf_2_CO_2_ carrier mobility.

Carrier type	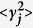	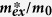	*E*1*x*	*E*1*y*	*Cx*	*Cy*	*μx*	*μy*
			(eV)		(Jm^−2^)		(10^3^ cm^2^V^−1^s^−1^)	
e	0.231	2.162	10.57	7.101	293.6	291.0	0.329	0.077
h (upper)	0.423	0.164	7.636	2.297	293.6	291.0	0.924	26.0
h (lower)	0.164	0.414	2.023	7.422	293.6	291.0	34.3	1.00

Carrier types “e” and “h” denote “electron” and “hole,” respectively. 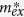 and 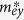 are the effective masses along the zigzag (*x*-) and armchair (*y*-) directions, respectively. *E*_1*x*_ and *E*_1*y*_ are the deformation potential constants, and *C*_*x*_ and *C*_*y*_ are the elastic moduli. *μ*_*x*_ and *μ*_*y*_ are the room-temperature carrier mobilities.
