# Supplementary material for: The thermal and electrical properties of the promising semiconductor MXene Hf2CO2
Source: Sci Rep. 2016 Jun 15;6:27971. doi: 10.1038/srep27971 (PMC4908405; doi:10.1038/srep27971)
Supplement: Supplementary Information [file srep27971-s1.pdf]

Supplementary information

The thermal and electrical properties of the promising semiconductor MXene Hf_2CO_2

Xian-Hu ZHA, Qing HUANG, Jian HE, Heming HE, Junyi Zhai, Joseph S. FRANCISCO, Shiyu DU *

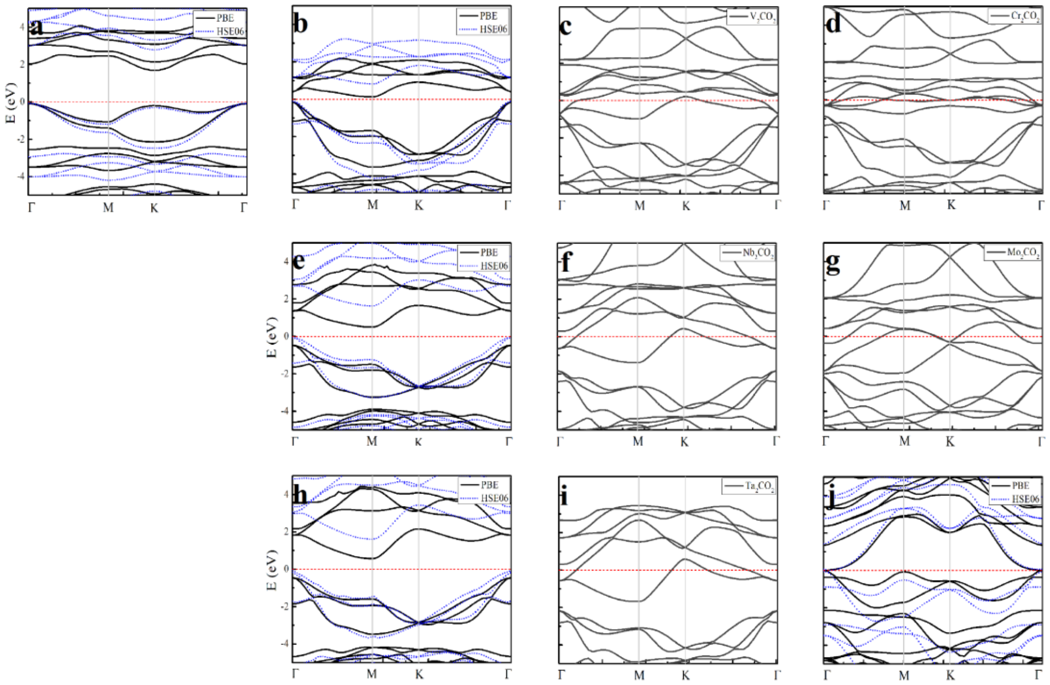

Figure S1 Electronic band structures of M_2CO_2 (M = Sc, Ti, V, Cr, Zr, Nb, Mo, Hf, Ta, W) MXenes. (a) Sc_2CO_2(b) Ti_2CO_2(e) Zr_2CO_2(h) Hf_2CO_2 (j) W_2CO_2 are semiconductors with indirect bandgaps, and others are metals. It should be noted that the Sc_2CO_2 and W_2CO_2 stable configurations are different from others[1,2].

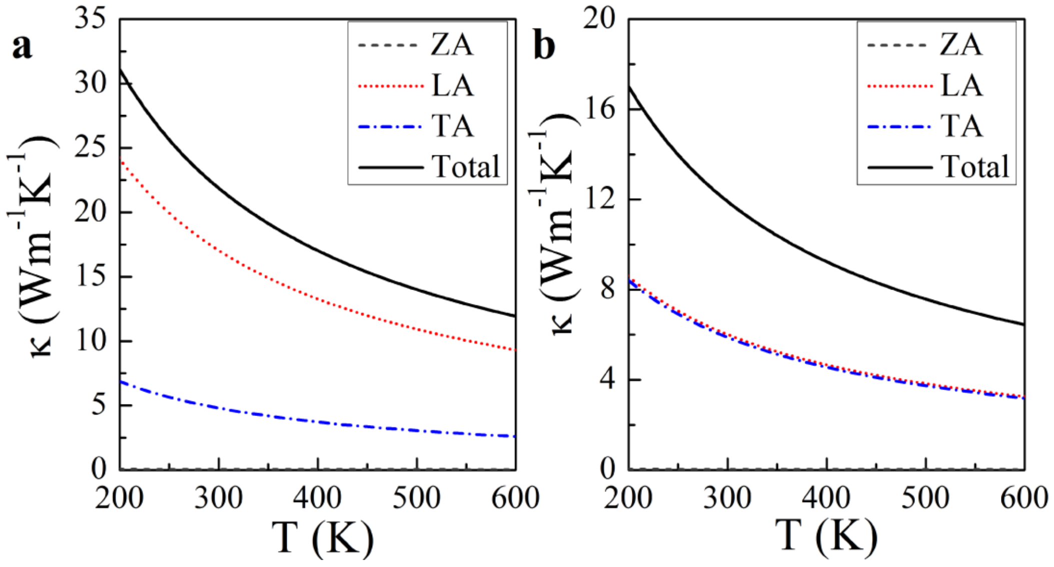

Figure S2 Temperature dependence of Ti_2CO_2 thermal conductivity. (a) Temperature dependence of Ti_2CO_2 thermal conductivity along the armchair direction ($d = 5\mu m$). The out-of-plane acoustic (ZA), longitudinal acoustic (LA) and transversal acoustic (TA) mode contributions to thermal conductivity are denoted in grey dashed, red dotted and blue dash-dotted lines, respectively. (b) Temperature dependence of Ti_2CO_2 thermal conductivity along the zigzag direction.

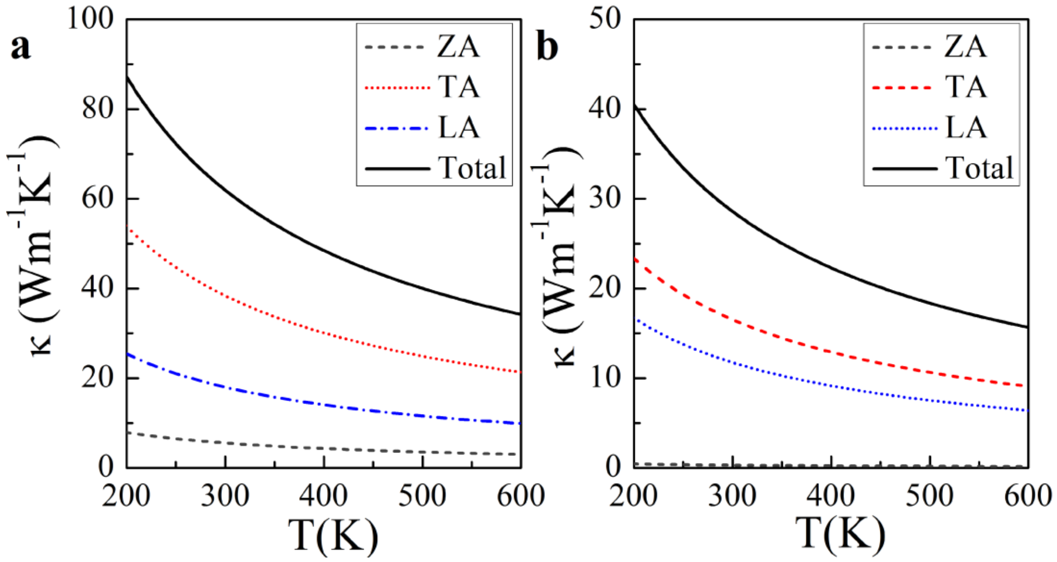

Figure S3 Temperature dependence of Zr_2CO_2 thermal conductivity. (a) Temperature dependence of Zr_2CO_2 thermal conductivity along the armchair direction ($d = 5\mu m$). The out-of-plane acoustic (ZA), longitudinal acoustic (LA) and transversal acoustic (TA) mode contributions to thermal conductivity are denoted in grey dashed, red dotted and blue dash-dotted lines, respectively. (b) Temperature dependence of Zr_2CO_2 thermal conductivity along the zigzag direction.

Table S1 Ti_2CO_2 carrier mobility

Carrier type	m_{ex}^*/m_0	m_{ey}^*/m_0	E_{1x}	E_{1y}	C_x (Jm^{-2})	C_y	μ_x (10^3 cm^{2}V^{-1}s^{-1})	μ_y
e	0.378	3.035	9.174	4.705	253.2	256.1	0.152	0.073
h(upper)	0.089	0.134	3.248	5.279	253.2	256.1	50.1	12.8
h(lower)	0.150	0.087	4.184	3.608	253.2	256.1	17.2	40.3

Carrier types "e" and "h" denote "electron" and "hole" respectively. m_{ex}^* and m_{ey}^* are the effective masses along zigzag (x-) and armchair (y-) directions, respectively. E_{1x} and E_{1y} are the deformation potential constants, and C_x and C_y are the elastic moduli. μ_x and μ_y are the room-temperature carrier mobilities.

Table S2 Zr_2CO_2 carrier mobility

Carrier type	m_{ex}^*/m_0	m_{ey}^*/m_0	E_{1x}	E_{1y}	C_x (Jm^{-2})	C_y	μ_x (10^3 cm^{2}V^{-1}s^{-1})	μ_y
e	0.265	1.873	13.78	5.210	265.2	262.2	0.152	0.149
h(upper)	0.164	0.380	9.840	1.798	265.2	262.2	1.37	17.5
h(lower)	0.362	0.163	5.452	6.043	265.2	262.2	2.08	3.71

Carrier types "e" and "h" denote "electron" and "hole" respectively. m_{ex}^* and m_{ey}^* are the effective masses along zigzag (x-) and armchair (y-) directions, respectively. E_{1x} and E_{1y} are the deformation potential constants, and C_x and C_y are the elastic moduli. μ_x and μ_y are the room-temperature carrier mobilities.

Reference:

1 Khazaei, M. *et al.* Novel electronic and magnetic properties of two-dimensional transition metal carbides and nitrides. *Adv. Funct. Mater.* **23**, 2185-2192, (2013).

2 Zha, X.-H. *et al.* Role of the surface effect on the structural,electronic and mechanical properties of the carbide MXenes. *EPL (Europhysics Letters)* **111**, 26007, (2015).
